# Using machine‐assisted topic analysis to expedite thematic analysis of free‐text data: Exemplar investigation of factors influencing health behaviours and wellbeing during the COVID‐19 pandemic

**DOI:** 10.1111/bjhp.70017

**Published:** 2025-09-11

**Authors:** Emma Ward, Felix Naughton, Pippa Belderson, Trisevgeni Papakonstantinou, Ben Ainsworth, Sarah Hanson, Caitlin Notley, Paulina Bondaronek

**Affiliations:** ^1^ Faculty of Medicine and Health Sciences University of East Anglia Norwich UK; ^2^ Department for Experimental Psychology University College London London UK; ^3^ Centre for Clinical and Community Applications of Health Psychology University of Southampton Southampton UK; ^4^ Institute of Health Informatics University College London London UK

**Keywords:** artificial intelligence, COVID‐19, health, machine learning, qualitative, thematic analysis, wellbeing

## Abstract

**Objectives:**

Investigate the use of machine learning to expedite thematic analysis of qualitative data concerning factors that influenced health behaviours and wellbeing during the COVID‐19 pandemic.

**Design:**

Qualitative investigation using Machine‐Assisted Topic Analysis (MATA) of free‐text data collected from a prospective cohort.

**Methods:**

Free‐text survey data (2177 responses from 762 participants) of influences on health behaviours and wellbeing were collected among UK participants recruited online, using Qualtrics at 3, 6, 12 and 24 months after the COVID‐19 pandemic started. MATA, which employs structural topic modelling (STM), was used (in R) to discern latent topics within the responses. Two researchers independently labelled topics and collaboratively organized them into themes, with ‘sense checking’ from two additional researchers. Plots and rankings were generated, showing change in topic prevalence by time. Total researcher time to complete analysis was collated.

**Results:**

Fifteen STM‐generated topics were labelled and integrated into six themes: the influences of and impacts on (1) health behaviours, (2) physical health (3) mood and (4) how these interacted, partly moderated by (5) external influences of control and (6) reflections on wellbeing and personal growth. Topic prevalence varied meaningfully over time, aligning with changes in the pandemic context. Themes were generated (excluding write‐up) with 20 h combined researcher time.

**Conclusions:**

MATA shows promise as a resource‐saving method for thematic analysis of large qualitative datasets whilst maintaining researcher control and insight. Findings show the interconnection between health behaviours, physical health and wellbeing over the pandemic, and the influence of control and reflective processes.

**Statement of contribution**

What is already known on this subject?

- AI is increasingly being used to analyse qualitative data to reduce the resources and time required for manual analysis of large qualitative data sets.
- Current methods of AI‐assisted analysis have strengths and weaknesses, with approaches that allocate full control to the AI having only moderate consistency with researchers.
- C‐19 pandemic research lacks qualitative/people generated perceptions of impacts over time.

What does this study add?

- MATA provides a hybrid machine and human analysis approach which maintains researcher control over thematic generation and interpretation.
- Findings identify key self‐reported factors shaping health behaviours, mood and wellbeing during the COVID‐19 pandemic using open‐ended responses, capturing lived experiences and a more granular view of pandemic impact.
- The prevalence of person‐generated factors shaping health behaviours, mood and wellbeing over a 2‐year period from the start of the C‐19 pandemic.

## BACKGROUND

Qualitative research is an established mainstay of psychological enquiry (Madill & Gough, 2008) and health research (Pope & Mays, 1995). Qualitative methodologies provide critical tools for investigating a range of ‘how and why’ research questions, many of which cannot be adequately answered using quantitative methodologies (Pope & Mays, 1995). In recent decades, technological advances have benefited quantitative enquiry and analysis substantially through improved software, processing power and modelling capability, enabling increasingly large and complex data sets to be interrogated. However, such technological advances have benefited qualitative research methods much less. While computer‐assisted qualitative data analysis software (CAQDAS) has existed for many years, ultimately qualitative analysis is undertaken manually, which is considerably time‐ and resource‐intensive (Nevedal et al., 2021).

Health care data is one of the fastest growing sources of data across all industries (Reinsel et al., 2018) and increasingly this includes qualitative data. Qualitative data can come from a variety of sources in addition to deliberate qualitative investigation, including health care records, health care service and technology survey data, patient recorded data, social media, etc. Furthermore, generative Artificial Intelligence (AI) agents or ‘chatbots’ have demonstrated the ability to undertake qualitative interviews using open‐ended questions via a digital interface (Juraimi et al., 2024). The scalability potential of using AI to collect qualitative data, alongside the large amounts of qualitative data being generated by health care services and other sources, highlights the increasing need to manage large qualitative datasets.

Large qualitative datasets are sometimes referred to as ‘Big Qual’ and have been defined as datasets that include data from more than 100 participants (Brower et al., 2019) or simply more qualitative data that an individual or small team can feasibly analyse manually (Weller et al., 2023). While there are epistemological questions about if and when such large datasets are required to adequately answer qualitative research questions (Vasileiou et al., 2018), Big Qual datasets are growing in number and volume, and will increasingly become a valuable source of insight to ultimately help improve health and healthcare. However, as it is not feasible to analyse Big Qual using manual analysis approaches, new analysis methods are needed (Weller et al., 2023).

Recent advances in the use of AI provide opportunities for enhancing the analysis of large qualitative datasets. Approaches, such as topic modelling (a type of natural language processing) (Leeson et al., 2019), supervised text classification (Smith & Tissing, 2018) and large language models (LLMs) (Prescott et al., 2024) have been investigated for undertaking or supporting qualitative analysis. Evidence to date indicates that the use of AI tools improves the efficiency and reduces the resource requirements for qualitative analysis compared to manual approaches (Cheligeer et al., 2022; Prescott et al., 2024; Towler et al., 2023). However, an important dimension is the extent to which the AI has full control of the final thematic or descriptive structure, and thus its reliability and authenticity, or is instead used for analysis augmentation.

Large language models (LLMs) show promise in qualitative analysis by identifying novel themes and theoretical links that human researchers may not identify (Hamilton et al., 2023; Wachinger et al., 2024). However, concerns about bias and low consistency with researchers suggest more research is needed before granting them full control (Hamilton et al., 2023; Kon et al., 2024; Peterson, 2024; Prescott et al., 2024). The use of AI to augment human‐directed analysis, rather than replace it, is an alternative that may circumvent some of the current limitations with LLM‐controlled qualitative analysis. Natural language processing (NLP) enhances the scalability and efficiency of text analysis, although on its own it lacks the depth and recognition of nuance and cultural contexts that humans bring to qualitative analysis (Weller et al., 2023). Using NLP to support, rather than control, qualitative analysis enables rapid AI‐identified concepts but with a layer of verification and ultimate control from the researcher.

Machine‐Assisted Topic Analysis (MATA) (Bondaronek et al., 2023; Towler et al., 2023) combines computational analysis with the expertise and insights of experienced qualitative researchers, leveraging both artificial and human intelligence to handle large volumes of text data. MATA employs structural topic model (STM), as proposed by Roberts et al. (2019), which discerns latent topics within texts. STM operates under the premise that documents are composites of topics, and it aids in extracting and delineating principal themes within a text corpus, mapping them onto individual documents. This method is particularly useful for systematically analysing extensive volumes of unstructured text.

One of the key strengths of STM lies in its incorporation of document‐level metadata into the topic modelling process (Roberts et al., 2019). This metadata can range from a document's creation date to the characteristics of its source (e.g., participants) and acts as covariates in the model. This integration allows for a nuanced estimation of the relationship between such variables and the discussed topics, enhancing the depth of analysis. It affects both the frequency of discussion of particular topics and the overall interpretive quality of the analysis. STM is instrumental for qualitative researchers, facilitating the identification of patterns and assisting in deriving deeper insights, interpreting and summarizing the topics. The output of STM is a series of topics that identify key patterns and categories from large datasets which can be interpreted and understood in context by the researchers to enable them to generate overarching themes from the data.

MATA was developed in response to the increasing need for rapid analysis of large quantities of free‐text data, a challenge that became especially evident during the COVID‐19 pandemic. For example, datasets like the UK NHS Test and Trace data (Bondaronek et al., 2023) where rapid actionable insights were crucial to improve the management of the pandemic. COVID‐19 impacts over time have been summarized quantitatively by cohort studies, including identifying health trends such as changes to health behaviours (Anyanwu et al., 2022; Bann et al., 2021; Herle et al., 2021), impacts of long COVID (Bowyer et al., 2023), initial increased mental health issues (Saunders et al., 2024) and widening health inequalities (Bann et al., 2021; British Medical Association, 2024; Finch & Tinson, 2022). However, there is a lack of examination of longitudinal qualitative cohort data, which could serve to contextualize quantitative cohort findings, provide additional insights and individual‐centred perspectives on pandemic impacts (Pope & Mays, 1995). One such dataset is the C‐19 Health Behaviour and Wellbeing Daily Tracker Study (Naughton et al., 2021). This study investigated how the pandemic affected health behaviour and wellbeing/mental health over time and included free‐text questions which hitherto were not able to be analysed due to resource limitations.

1. This paper has two parallel aims. Firstly, it aims to describe the process of applying MATA for analysing qualitative free‐text data in a large longitudinal dataset. Secondly, it describes the application of MATA to answer the question of which factors were self‐reported to influence health behaviours, mood and wellbeing over a two‐year period since the COVID‐19 pandemic started, among participants of the C‐19 Health Behaviour and Wellbeing Daily Tracker Study (Naughton et al., 2021). This second aim serves as both a demonstration of the method and an investigation into this under‐investigated research question. Objectives relating to this second aim are: Explore factors (as thematic topics), reported in free‐text responses at 3, 6, 12 and 24 months follow‐up, that are perceived to influence health behaviours, mood and wellbeing.
2. Explore the prevalence of identified factors at each follow‐up timepoint and change in prevalence over time.

## METHODS

### Design and participants

Qualitative investigation of free‐text data collected in follow‐up surveys from a prospective cohort of UK residents recruited in April 2020.

Participants were 18 years and above, living in the United Kingdom, with access to a smartphone. Participants were recruited online and were purposively sought to include people with a high‐risk physical condition for COVID‐19 (in line with UK national definitions in 2020), those living in an area of high deprivation and those with a self‐reported mental health issue. Recruitment was via social media and targeted to vulnerable groups (e.g., women's groups, mental health support groups).

### Procedure

Once enrolled and after completing the baseline questionnaire and a 12‐week measurement period of daily ecological momentary assessments (Naughton et al., 2021), all participants were invited to complete follow‐up surveys at 3, 6, 12 and 24 months post‐baseline. Surveys were hosted on Qualtrics XM software. These online surveys were prompted by text message, with several reminders and collected data on COVID‐19, health behaviour and wellbeing/mental health‐related measures. For further details about the parent study, see the project Open Science Framework page (https://osf.io/dm853/) and the wave 1 findings article (Naughton et al., 2021).

At the end of each follow‐up survey, participants were invited to answer a free‐text question inviting them to ‘Please… tell us what factors have influenced your health behaviours, mood and sense of wellbeing over the last [3/12] months’. The 3, 6 and 12 month survey enquired about the last 3 months and the 24 month survey about the last 12 months. The responses to this free‐text data are the focus of this article. The follow‐up surveys were conducted at four key timepoints over a 21‐month period during the COVID‐19 pandemic. The 3‐month survey was collected in July 2020, when England was beginning to emerge from the first national lockdown. The 6‐month survey was collected in October 2020, as COVID‐19 cases were rising and local tiered restrictions were introduced. The 12‐month survey was collected in April 2021, shortly after England had exited a second lengthy national lockdown. Finally, the 24‐month survey was collected in April 2022, following gradual lifting of all remaining restrictions.

### Machine‐assisted topic analysis (MATA)

Structured data, comprising participant identifier, timepoint, age, gender, deprivation, COVID‐19 risk factors and the prevalence of mental health issues, were included in the models as covariates to account for their potential influence on the outcomes. These variables provided a foundation for the analysis, ensuring that key demographic, socioeconomic and health‐related factors were considered systematically.

Data pre‐processing was conducted using R (version 3.5.2) and included the following steps:

1. Cleaning free‐text responses: Free‐text data were handled using a combination of base R functions and the quanteda (version 2.0.1) and STM (version 1.3.3) packages. Initial cleaning involved removing punctuation, symbols and numbers from the text, ensuring consistency and preparing it for further processing.
2. Removing incomplete or redundant data: Observations containing missing values or duplicate entries were identified and removed to maintain the integrity of the dataset.
3. Tokenization of text: The cleaned free‐text responses were converted into tokens—individual words—using the quanteda package. Tokenization facilitated the transformation of textual data into a structured format suitable for analysis.
4. Eliminating stop words: Commonly used words with minimal analytical value, such as ‘and’ or ‘the’, were removed to focus on more meaningful content.
5. Stemming tokens: Words were reduced to their root forms through a process called stemming. This step acted as a normalization process, reducing variability in the text and decreasing the size of the dictionary.

### Structural topic modelling of the free‐text data

We employed the STM as our topic modelling method (Towler et al., 2023). Diagnostic analysis was conducted prior to running the models to identify the optimal number of topics. This process involved balancing semantic coherence and exclusivity, guided by relevant metrics and the specific aims of the analysis.

To identify the best model configuration, we tested models with 5 to 40 topics, incorporating the structured data reported above as covariates. We evaluated the models based on their semantic coherence scores (Mimno et al., 2011) and residuals. Following a visual examination of diagnostic plots (Appendix S1), we determined that a model with 15 topics was optimal for the analysis. To facilitate qualitative analysis, we extracted 20 representative ‘quotes’ for each topic from the dataset.

### Researcher interpretation of topics and generation of themes

The aim of the researcher‐led qualitative analysis was to interpret the output, agree upon narrative labels for the topics and organize topics into a broader thematic structure, identifying factors as outlined in Objective #1.

We analysed the STM in five stages:

1. EW and FN independently categorized the machine output (in Excel) by writing short descriptive notes for each quote within a topic and then reviewing the notes for similarities of meaning to identify topic meaning.
2. EW and FN then met to compare their independent interpretation of machine output, resolve any disagreements in the interpretation through discussion and agree the draft MATA code labels for each topic.
3. EW and FN worked together to manually organize the MATA code labels into broader themes.
4. PB and SH separately reviewed the text analysis and labels to ‘sense check’ the interpretation. EW, FN, PB and SH met to discuss the MATA code labels and broader theme labels and structure, agreeing on a consensus of interpretation through collaborative discussion.
5. EW conducted an analytical write‐up of the qualitative analysis which consisted of re‐reviewing free‐text responses provided in each topic in the context of the researchers' interpretation of topic meaning. The write‐up was reviewed by the other researchers. As is typical in qualitative research, this stage was an iterative process and resulted in some changes to label headings and thematic structure. Changes were agreed by the team and summarized in the findings section.

### Prevalence of topics by timepoint

To meet objective #2, exploratory analysis was undertaken on the prevalence of topics at each follow‐up timepoint and change in prevalence over time. The prevalence of each user response to each topic was averaged to calculate a mean topic weighting for each follow‐up timepoint (3, 6, 12 and 24 months; the higher the mean weighting score indicating greater prevalence at that time). How the prevalence of topics changed over time in comparison to each other was explored by plotting topic prevalence at each time point using line graphs. Prevalence scores were converted to rankings for each timepoint for ease of interpretation (1 = most prevalent through to 15 = least prevalent).

## FINDINGS

The characteristics of the sample are shown in Table 1.

**TABLE 1:** Baseline sample description (*N* = 762).

Characteristic	%^a^	*N* ^b^
Age category (years)		
18–24	7.6	58
25–44	32.4	246
45–64	46.0	349
65+	14.0	106
Gender		
Male	25.4	191
Female	74.6	562
Ethnicity		
White	96.3	731
Other	3.7	28
Number of adults in household		
1 (living alone)	21.2	161
2	57.8	439
3	13.7	104
4+	7.5	56
Keyworker		
Yes	26.8	204
No/not working	73.2	558
Employment status		
Employed/self‐employed/freelance	60.2	459
Not working (student/home carer/retired)	23.9	182
Never worked or long‐term unemployed	.3	2
Unemployed and looking for work (not due to COVID‐19)	1.6	12
Out of work/furloughed/leave of absence (due to COVID‐19)	10.6	81
Unable to work because of sickness or disability	3.4	26
Index of Multiple Deprivation (IMD) quintile (1 = most deprived)^c^		
1	11.4	85
2	15.8	118
3	25.0	187
4	22.6	169
5	25.3	189
COVID‐19 at risk health condition		
Very high‐risk health condition	7.2	55
Increased risk health condition	24.5	187
No increased risk health condition	68.2	520
Self‐reported mental health issue		
Yes	5.8	44
No	94.2	718

^a^ Percentages may not add up to 100 due to rounding.

^b^ Due to missing data, totals may not add to 1044.

^c^ Combined using IMD decile scores from England (2019), Northern Ireland (2017), Scotland (2020) and Wales (2019).

### Total researcher time to complete analysis

Stages 1–4 of the researcher interpretation took approximately 20 h of combined researcher time, with Stage 5 (write‐up) taking approximately 28 h.

### Self‐reported influential factors during the pandemic

**FIGURE 1:**
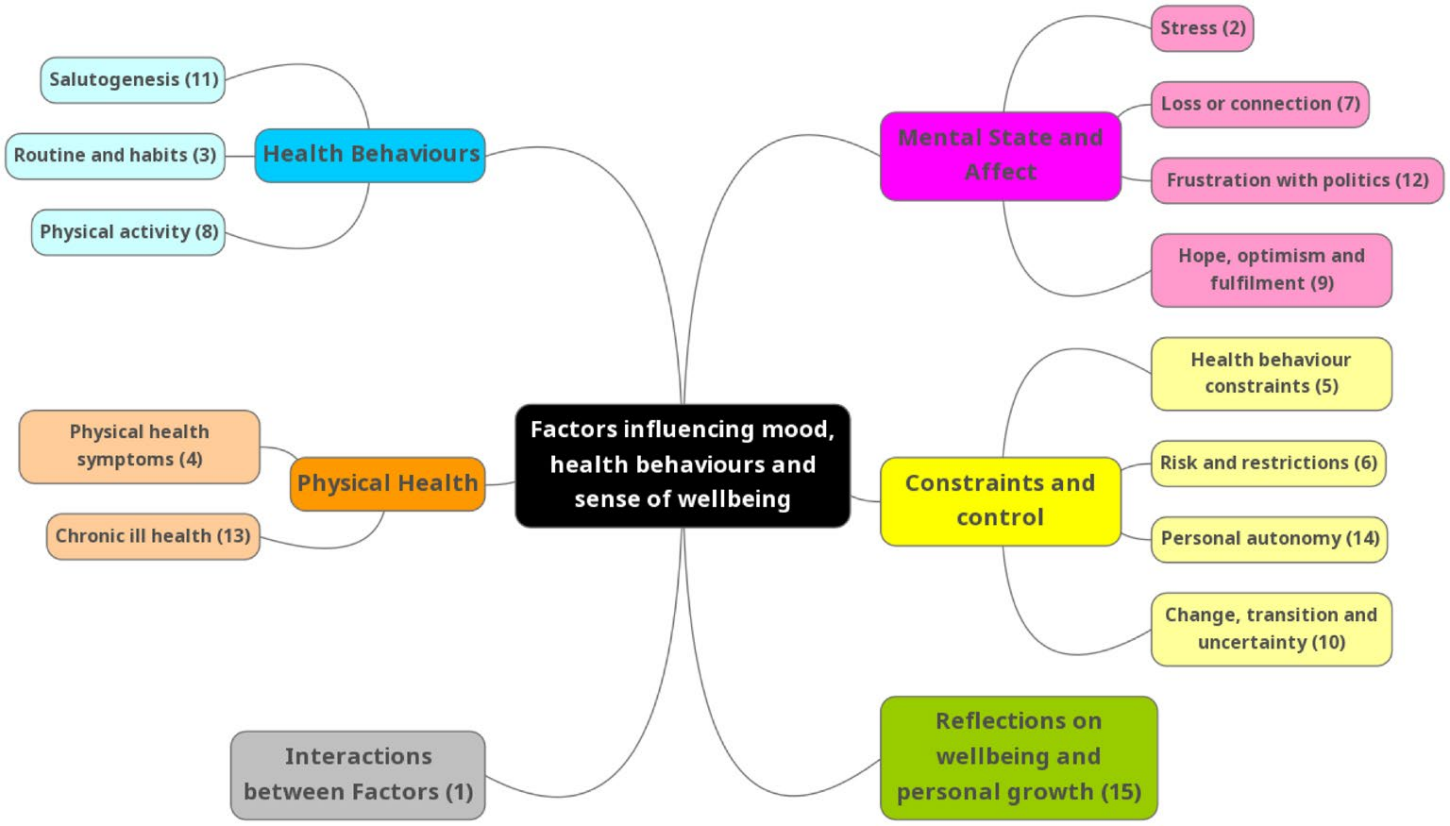
Thematic map of topic labels clustered into broader themes.

The final thematic structure of the researcher interpretation of the 15 machine‐generated topics from free‐text responses is shown in Figure 1, showing thematic clusters of topics resulting in six broader themes. Table 2 defines each topic and provides example quotations, summarizing the core meaning of each topic and outlining what differentiates the topics, rather than an attempt to describe everything within the topic.

**TABLE 2:** Topic labels, thematic group and descriptions with example quotes.

Topic label	Topic thematic group	Topic description	Topic example quote
Salutogenesis	Health behaviours	Recognizing self‐care behaviours that improved health and wellbeing, predominantly sleep, healthy diet, exercise, fulfilling hobbies and activities, and positive social connection with friends/family.	‘Video calls with my family and friends. Plenty of sleep. No unexpected visitors. Mostly good weather. Online connection to my church. Time to cook and eat well. Kindness of my colleagues at work especially my managers’
Routines and habits	Health behaviours	Building positive routines and habits to undertake health behaviours. Low mood and external constraints (e.g., lockdown, weather) were identified as hindering healthy habits, with some struggling against unhealthy temptations. Efforts to recalibrate and then plan for future positive behaviour change discussed.	‘At the beginning of lockdown, I was already drinking more than I should. After lockdown started my levels of drinking increased. I decided I'd had enough of drinking on [date], so gave up drinking on that date, except for the occasional beer. Unfortunately, after the clocks changed, I started drinking wine again and, since then, have been drinking wine daily. I will try to give up again in the near future and I will keep trying until I succeed’.
Physical activity	Health behaviours	Positive impacts of physical activity on wellbeing such as destressing, socializing and providing a sense of achievement. Barriers included physical health issues, low motivation and pandemic‐related constraints, while facilitators included time availability, access to the outdoors and dog ownership	‘Being able to go to the gym has been an enormous help. Good weather for my bike ride too’
Physical health symptoms	Physical health	Physical health symptoms (mostly musculoskeletal, but also respiratory, dental and hormonal related symptoms). Consequences such as fatigue, weight changes and chronic pain. Self‐directed treatment (e.g., gentle exercise) discussed in some quotes, whilst others covered frustration with delays to health care with private care being sought as a result	‘I am currently suffering with Frozen Shoulder on my right shoulder. Painful to sleep and discomfort to raise arm above head’
Chronic ill health	Physical health	Impact and concern brought about by living with ‘chronic ill health’, primarily long COVID, although other conditions featured. Limitations in mobility, cognition and mental health, with heightened anxiety over COVID risks, access to health care, financial insecurity and general uncertainty about the future were all reported	‘I have a number of long COVID difficulties: Fatigue, breathlessness like breathing through a blanket. Painful eyes, lips and skin rashes. Can't breath through my nose due to pain. Aching arms, hands and feet. Occasional flu‐like symptoms. All of these present together/separately and generally each day. I do not have a day without symptoms. This has caused my usually very busy life to close‐down. I isolate myself and feel there will be no future without this, or at all. I feel that 6 months after initial diagnosis of COVID, I may not ever return to good health’
Stress	Mental state and affect	Increased ‘stress’, predominantly work and family‐related (but also environmental for example, problem neighbours, moving house), triggering feelings of pressure, anxiety and exhaustion. Juggling stressors without respite, led to feelings of failure and guilt. Some described stress being compounded by perceived lack of support from employers or partners	‘Lack of childcare to enable working. Utterly exhausted by having to juggle work and homeschooling’
Loss and connection	Mental state and affect	Most quotes discussed bereavement. Pandemic restrictions also featured and were described as resulting in loss of social connection or fulfilment in life. Negative impacts on mood, confidence, motivation and sense of identity were reported. Some quotes, however, described finding solace in new roles or rekindling social connection and activities post‐restrictions	‘I live alone. I have followed the guidelines and therefore usually see no one apart from 5 work colleagues when I go to office 1 day a week…. Possibly because I'm 65 and have hardly driven anywhere for 12 months I am now anxious at thought of driving a few miles —pre COVID I'd have driven 600 miles to see son in [Scottish city] and not given it another thought—I travelled through [Asian country] on my own 2 years ago—the isolation has knocked my confidence’
Frustration with politics	Mental state and affect	Frustration primarily with the government's perceived incompetence regarding pandemic management. Frustrations extended in some quotes to broader political issues like Brexit, corruption, conflicts and ‘culture wars’. Some participants expressed anxiety about the future, fearing decline in quality of life and global stability. A few quotes described a sense of mistrust in decision makers, exacerbated by perceived political failures and irresponsible media reporting.	‘The government's poor handling and lack of accountability, and in particular the Cummings saga had the biggest detrimental impact on my wellbeing in the whole of lockdown’
Hope, optimism and fulfilment	Mental state and affect	Discussions on taking specific action to improve mental state such as seeking support, implementing routines and new activities, and setting goals and plans. External circumstances such as change in season, lockdown easing and vaccinations, were described as contributing to these positive feelings and there were expressions of gratitude. Some quotes, however, also included negative feelings and factors influencing those.	‘Being vaccinated. Spring coming & a sense that things are improving for everyone. COVID rate going down—My family are being pretty stoic about when we can all get together again. We have been very lucky to have not lost any close family members to it. Have been content with what I have on the whole which I know is much more than many people have. I did manage to do Dry January which was affirming that I can stop drinking should I chose’
Health behaviour constraints	Constraints and control	Perceived lack of control regarding factors impacting on their ability to engage in healthy behaviours. Factors including limiting health conditions, COVID anxiety, lack of time due to caring and/or work responsibilities and changes in living circumstances. Facilitators discussed as increasing a sense of control were easing of pandemic measures, online communication, support and medication.	‘COVID fatigue and adverse reaction to the booster (heart inflammation) finally resolved and I could get back to exercising’
Risk and restrictions	Constraints and control	Perceptions of COVID ‘risk and restrictions’. COVID risk was perceived to be increased due to health conditions, COVID rule‐breakers and Governmental mishandling. To reduce risk, participants discussed following COVID rules and self‐imposed restrictions such as avoiding public spaces. To reduce COVID anxiety, some participants discussed information seeking or, conversely, avoiding media. Anxiety was also expressed in relation to ability to work or being separated from friends/family due to restrictions.	‘Disregard by the general population that they will keep to the rules. It scares me. I have stayed in my family bubble and rarely gone to the public domain. Would turn if I felt a place was too crowded’
Personal autonomy	Constraints and control	Lack of control over ‘personal autonomy’, resulting in reduced motivation and feelings of loneliness, boredom and anxiety. Threats to personal autonomy included the pandemic measures (featured in most quotes), but also the increased perception of the world becoming a riskier place. This was reported as being due to COVID rule‐breakers prioritizing their own autonomy over risk to others, overexposure to media and news consumed by COVID and other negative news stories, and the perception that people were generally becoming more intolerant.	‘When in total Lockdown felt more in control than I do now—thought there would be a definite end and sense of closure to things. Now still social distancing and things still not back to normal. Still can't hug people and meet for proper large family gatherings, etc’
Change, transition and uncertainty	Constraints and control	Changes were predominantly in relation to work or education, although some quotes discussed changes such as moving house or relationship changes. Many changes were reported as being out of the participants' control. Both positive and negative effects were described such as impacts on mood, financial security and stability. A few quotes discussed anxiety due to changes in work practices leading to greater exposure to COVID.	‘Having graduated in July, job hunting and the prospect of a relationship breakdown, have greatly impacted all’
Interactions between factors		A standalone theme about the interactions between factors including health behaviours, physical health, mental state and external constraints. The interactions between these factors had negative, mixed or positive impacts. A common example of negative impacts was that experiencing ill health (physical health) and waiting for treatment (external constraint), led to low mood (mental state). An example of a mixed interaction, prevalent in a few of the quotes, was that easing of lockdown (external constraint) meant that participants were able to exercise/socialize more (health behaviour), but had anxiety doing so (mental state). There were also examples of feedback loops, for example, low mood (mental state) leading to overeating (health behaviour) leading to increased low mood. Another example was developing motivation to exercise (mental state), then exercising (health behaviour), leading to improved mood.	‘I have had frequent UTIs, blood markers have shown up showing abnormal liver function and now CA125 levels are high. I am waiting a pelvis scan to check for ovarian cancer, this is affecting my mindset and energy levels at the moment’ ‘The easing of restrictions has made me a lot more willing to be active however also nervous and anxious due to not knowing how the outcome will be’
Reflections on wellbeing and personal growth		A standalone theme about reflections of the lockdown measures on wellbeing and personal growth. Many quotes referenced lockdown being a quieter, slower or less demanding time, resulting in stronger relationships and sense of community, pursuit of interests, enjoyment of nature, starting new challenges and developing a sense of gratitude. Personal revelation and appreciation about a sense of importance in life featured in many of the quotes (e.g., accepting a dislike of socializing, appreciating the simplicity of having less choice). Other quotes discussed barriers to personal growth.	‘COVID hasn't bothered me one bit. I've thoroughly enjoyed every minute of lockdown. Far from diminishing my quality of life it has enhanced it wonderfully. What I've learned from all this is that I'm not really someone who enjoys socializing. I'm far happier at home with my wife and cat. Socializing is more stressful than prior to COVID I realized’

### Main theme narrative meaning and topic prevalence

**FIGURE 2:**
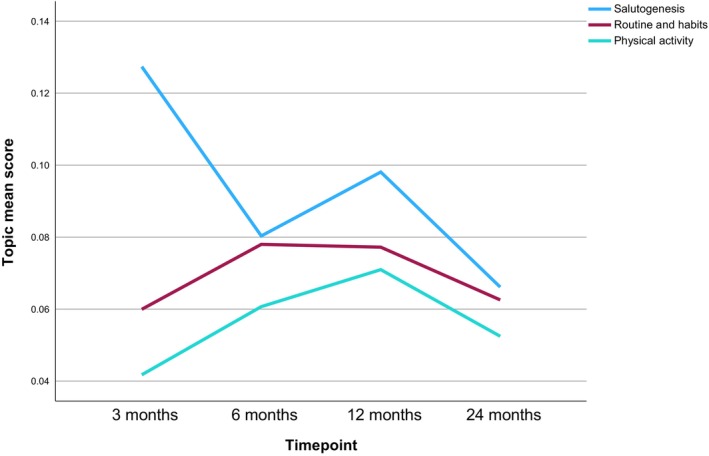
Health behaviour broad theme topic mean score by timepoint.

**FIGURE 3:**
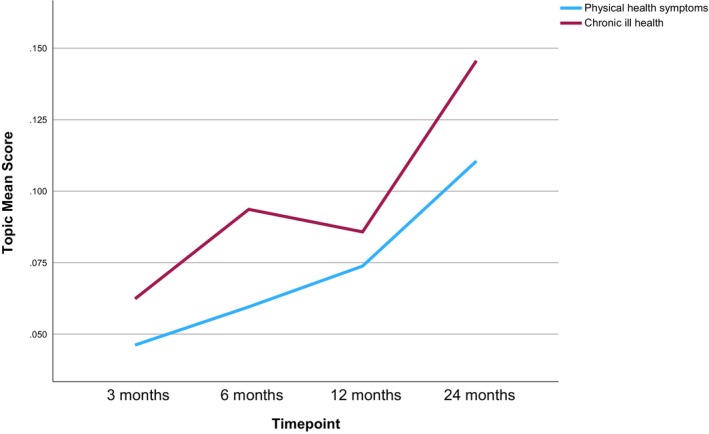
Physical health broad theme topic mean scores by timepoint.

**FIGURE 4:**
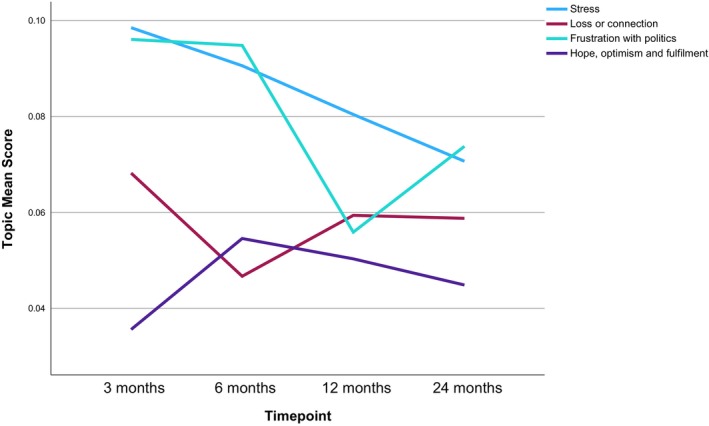
Mental state and affect broad theme topic mean scores by timepoint.

**FIGURE 5:**
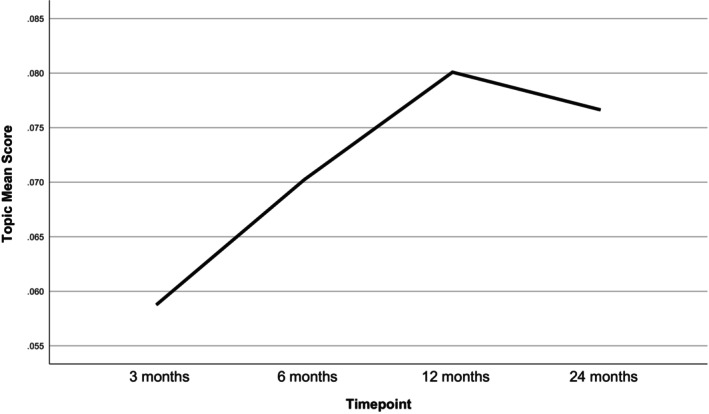
Interactions between factors topic mean score by timepoint.

**FIGURE 6:**
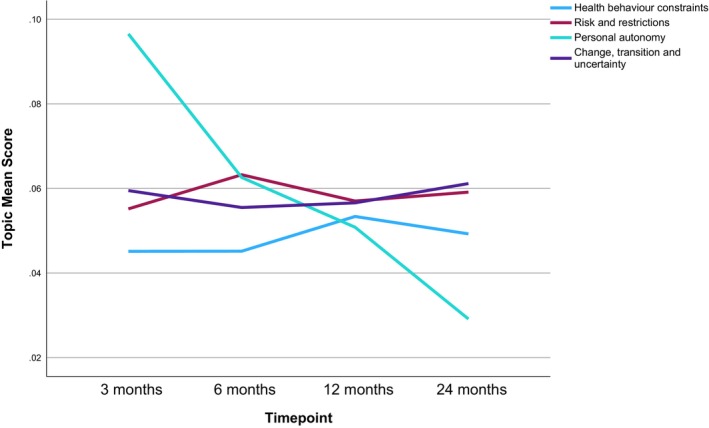
Constraints and control broad theme topic mean scores by timepoint.

Six main themes were manually derived from the analysis of the 15 topics and are narratively summarized below with topic labels in italics and illustrative quotes. Topic prevalence in relation to each timepoint is discussed below, structured by main themes, and shown in Table 3 and Figures 2, 3, 4, 5, 6.

**TABLE 3:** Scores and rankings for each topic by timepoint.

Topic	Theme	3 months		6 months		12 months		24 months	
		Score	Rank	Score	Rank	Score	Rank	Score	Rank
Salutogenesis	Health behaviours	.1274	1	.0803	4	.0981	1	.0661	6
Routines and habits	Health behaviours	.0600	7	.0780	5	.0772	5	.0626	7
Physical activity	Health behaviours	.0417	14	.0607	9	.0710	7	.0525	11
Physical health symptoms	Physical health	.0462	12	.0595	10	.0738	6	.1105	2
Chronic ill health	Physical health	.0624	6	.0936	2	.0857	2	.1456	1
Stress	Mental state and affect	.0985	2	.0906	3	.0804	3	.0707	5
Loss and connection	Mental state and affect	.0682	5	.0467	13	.0594	8	.0587	10
Frustration with politics	Mental state and affect	.0960	4	.0948	1	.0559	11	.0738	4
Hope, optimism and fulfilment	Mental state and affect	.0356	15	.0545	12	.0503	14	.0449	13
Health behaviour constraints	Constraints and control	.0451	13	.0452	14	.0534	12	.0493	12
Risk and restrictions	Constraints and control	.0552	10	.0632	7	.0570	9	.0591	9
Personal autonomy	Constraints and control	.0965	3	.0626	8	.0508	13	.0292	15
Change, transition and uncertainty	Constraints and control	.0595	8	.0555	11	.0566	10	.0612	8
Interactions between factors		.0588	9	.0702	6	.0801	4	.0766	3
Reflections on wellbeing and personal growth		.0490	11	.0445	15	.0503	15	.0394	14

#### Health behaviours

This theme relates to participants' identification of healthy behaviours perceived as important to their sense of wellbeing, promoting *salutogenesis*, (particularly *physical activity*, but also behaviours such as sleep, healthy eating, hobbies and social connection) and their ability to establish or recalibrate plans, *routines and habits* to support the behaviours. Barriers to undertaking health behaviours included low mood, physical health issues, external constraints (e.g., pandemic measures), while facilitators included time, resources, access to outdoor spaces and dog ownership. Aspects of this theme are illustrated in the quote below:

Being on reduced hours at work, and being static and not checking tickets, not walking through the train, meant I needed to up my exercise to help lose weight. The walks/jogs have made me happier, healthier and done wonders for me mentally. Only downside not being able to see my 2 teenage daughters, but we have talked and spoke online every few days and every day I get closer to seeing them. (Routines and Habits)

*Salutogensis* was the most prevalent topic at 3 and 12 months (Table 3, Figure 2), just after the strictest lockdown periods. *Routines and habits* showed relatively consistent prevalence across the time points; 5th most prevalent topic at 6 and 12 months and 7th at 3 and 24 months. *Physical activity* was the 14th most prevalent topic at 3 months but had increased in prevalence at the other time points; 9th, 7th and 11th, respectively.

#### Physical health

Participants described *physical health symptoms* (e.g., musculoskeletal, respiratory) or *chronic ill health*, with long COVID featuring in many responses. Physical health was described as impacting mental health and ability to undertake activities, including health behaviours. Problems accessing health care and delays to treatment were a source of frustration, with some participants describing turning to the private sector as a result. This sense of frustration is evident in this quote:

Long waiting list for apparently urgent surgery (15 months so far) has increased my depression as well as my chronic pain, as well as exacerbating my other chronic illnesses (fibromyalgia, CFS, IBS, migraines). Worsening health has led to increased need for rest and time in bed, missing out on lots of things‐ including the ability to work, affecting my wages, increasing my anxiety as I am struggling to pay bills. All because of the length of the waiting list for gynaecology surgery. (Chronic ill health)

Twenty‐four months post‐lockdown, both of the topics within this theme demonstrated the highest prevalence of all topics (Table 3, Figure 3). The prevalence of *physical health symptoms* rose sharply at each timepoint (ranked 12th/10th/6th/2nd respectively), and similarly *chronic ill health* started as 12th most prevalent at 3 months, then rose sharply to 2nd (6 and 12 months) and then 1st (24 months) most prevalent.

#### Mental state and affect

Participants reported influences on mental states. External influences included *stress*‐filled environments, predominantly related to managing work and family, which resulted in feelings of exhaustion, failure and guilt. Lockdown measures preventing social connection and experiencing bereavement were described as generating feelings of *loss* and grief. Perceived governmental misgovernance and incompetencies influenced feelings of *frustration with politics* and anxiety about the future:

The useless way this government has handled this pandemic, the useless track and trace system which is the key to getting us out of this clusterf*ck, and the billions of billions of handouts to cronies, impact on my mental health, as do the treatment of refugees, BLM issues. Hard to stay positive. (Frustration with politics)

Positive influences on mood included easing of pandemic measures, vaccinations and changing seasons/weather, which were described as generating feelings of *hope, optimism and fulfilment*. Participants also discussed self‐directed influences such as support seeking and engaging in health behaviours.

As shown on Table 3 and Figure 4, *stress* was among the top‐third most prevalent topics at all timepoints, with a slight reduction in prevalence over time (2nd/3rd/3rd/5th respectively). *Loss and connection* was the 5th most prevalent at 3 months and then fluctuated between the bottom and middle‐third at the other timepoints (13th/8th/10th respectively). *Frustration with politics* was the 4th most prevalent topic at 3 months, then was most prevalent at 6 months before dropping to 11th most prevalent at 12 months and then back up to 4th at 24 months. *Hope, optimism and fulfilment* was the least prevalent topic at 3 months and then remained in the bottom‐third of topics for the other timepoints (12th/14th/13th, respectively).

#### Interactions between factors

*Interactions between factors* is a main theme consisting of a standalone topic. Participants explicitly reported interactions between all or some of the factors identified in the above three themes. Interactions between health behaviours, physical health and mental state, modified by external constraints, could have positive, negative or mixed impacts. For example, a common mixed impact was improved physical health but increased COVID anxiety because of engagement in outdoor physical activity instigated by pandemic measures easing. The quote below is an example of a feedback loop showing the positive impact of treatment on mood which enabled healthy behaviours which further improved mood:

I started medication and therapy for my anxiety and depression episode which started in the summer. Since then, I've felt more motivated to look after my health (diet and exercise) and have been feeling more optimistic about the future. I've been feeling much less anxious on a daily basis.

Table 3 and Figure 5 show that the *interactions between factors* topic increased in prevalence over the study period. It moved from 9th most prevalent at 3 months to 6th at 6 months, 4th at 12 months and 3rd at 24 months.

#### Constraints and control

Participants reported influences on their perceived sense of control over *personal autonomy*, ability to undertake *health behaviours*, and *change, transition or uncertainty* relating to work or personal circumstances. Constraints included limiting health conditions, lack of time, pandemic measures and COVID *risk and restrictions*. Regarding perceived COVID risk, prevalence of COVID ‘rule‐breakers’, exposure to negative media reporting, work practices increasing COVID exposure and perceived government mishandling increased the perception of risk, resulting in COVID anxiety. The easing of pandemic measures, online communication and accessing support/treatment was discussed as increasing one's sense of control. Paradoxically, constraining one's freedom was also seen as a way to gain control amidst the uncertainty of the pandemic:

Sense of safety for not having to leave the house every day; avoiding public crowds or busy places. (Risks and restrictions)

*Personal autonomy* reduced in prevalence over time from 3rd most prevalent at 3 months, then 8th at 6 months, 13th at 12 months and least prevalent (15th) at 24 months (Table 3, Figure 6). *Risk and restrictions* were consistently in the middle‐third most prevalent topics across timepoints (10th/7th/9th/9th respectively). *Health behaviour constraints* were consistently in the bottom‐third most prevalent topics (13th/14th/12th/12th respectively). *Change, transition and uncertainty* were also relatively consistent over time, ranking between 8th to 11th most prevalent (8th/11th/10th/8th respectively).

#### Reflections on wellbeing and personal growth

*Reflections on wellbeing and personal growth* is a main theme consisting of a standalone topic. Some participants reported that the pandemic was a quieter, less demanding time that allowed for personal growth:

I've LOVED the lockdown. It's been enormously enjoyable and I rather miss it now it's over. I've loved the quietness, the peace, the absence of annoying things like sport, trashy TV stuff and big events. I've loved being free from the ‘let's go out, meet friends and have fun’ sort of thing that happens in life. I've loved the absence of traffic, how stress‐free shopping has been. How much calmer and more relaxed everyone else has been. I've been amazingly busier than ever with my own interests. I've had a sense of fulfilment and achievement from all that.

Some participants discussed the strengthening of relationships, fostering community, encouraging hobbies and starting businesses. Participants reflected on life's priorities, embracing simplicity and developing gratitude. Barriers to reflection and personal growth (such as time) were also discussed by some.

**FIGURE 7:**
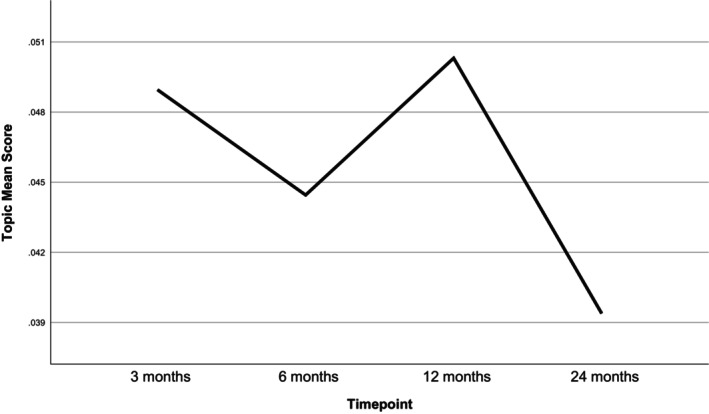
Reflections on wellbeing and personal growth topic mean score by timepoint.

At 3 months, this topic was the 11th most prevalent and for the other timepoints prevalence decreased to 15th at 6 and 12 months and 14th at 24 months (Table 3, Figure 7).

## DISCUSSION

MATA uses machine learning to identify patterns within large textual datasets, grouping data into topics, which are subsequently interpreted for meaning by researchers. Rather than a ‘human in the loop’ approach, MATA facilitates a ‘human in control’ approach over the analytic process, using AI to enhance efficiency while preserving the ‘qualities’ (e.g., depth and contextual understanding) essential to qualitative research. This paper's first aim was to describe the process of applying MATA to qualitative free‐text data, collected as part of the C‐19 Health Behaviour and Wellbeing Daily Tracker Study (Naughton et al., 2021). MATA was an efficient approach to analyse the large qualitative dataset. All 15 topics were able to be interpreted by the research team, reinforcing this number of topics as optimal, whereas a previous study using MATA generated ‘non‐sense topics’, which researchers were unable to interpret meaning (Towler et al., 2023).

As with previous studies employing MATA (Bondaronek et al., 2023; Towler et al., 2023), researcher time was considerably reduced, especially relating to the coding, theme generation and conceptualization stages of thematic analysis (Braun & Clarke, 2006). The reduction in time directly resulted from researchers having to read and interpret only 300 ‘best fit’ free‐text responses (20 per topic), as opposed to all 2172 responses. In addition, the machine generation of the 15 topics meant that the researchers bypassed the time‐consuming inductive coding stage of manual thematic analysis and instead undertook a quicker process of ‘reverse coding’ to interpret the conceptual meaning of topics (Stage 1 in our procedure). The time taken to complete the analytic write‐up after theme generation (Stage 5 in our procedure), which was conducted by a researcher without machine input, was not reduced and was comparable to the time usually taken in manual qualitative analysis. This stage, however, was particularly important to the interpretation process for this study, given the machine rather than researcher generated the initial topics. It allowed for researchers to check for contextual accuracy, investigate the deeper meaning of the topics and differences between them and develop a coherent narrative about the themes using example data.

The use of AI for qualitative analysis is increasing and this can create risks. One example risk is the quantification of qualitative data. This could risk oversimplifying findings by overlooking outliers, nuanced responses and systemic influences such as broader social determinants. Balancing efficiency with depth and maintaining contextual understanding remains a critical challenge. We argue that MATA may help to mitigate concerns about the quality of AI‐driven models in analysing participants' experiences while maintaining the depth and contextual understanding associated with qualitative research, as the process of human analysis was designed to be both collaborative and transparent.

The second aim of the paper was to explore which factors were reported to influence health behaviours, mood and wellbeing during the COVID‐19 pandemic. Six main themes were identified by researchers by interpreting the 15 topics for conceptual meaning and then clustering topics into themes. Themes were split into three key areas involved in contributing to pandemic impacts relating to behaviour, health and wellbeing, and three higher‐order themes relating to the complex interactions of those key areas, and how constraints and control and reflections on wellbeing and personal growth could modify impacts. The main themes align with and complement previously published qualitative findings derived from our parallel studies on the same longitudinal cohort, which used established qualitative methodologies (interviews, photo elicitation and thematic analysis) (Hanson et al., 2023; Notley et al., 2022). The key themes we identified in these other qualitative studies, namely disruption, adaptation, loss and salutogenesis, are evident as equivalent MATA topics/themes, enhancing validity across study elements via triangulation of data sources and methods. In addition, similar themes related to pandemic health and wellbeing impacts, health behaviours and coping strategies and adaption have been identified in the wider COVID qualitative literature, albeit with smaller samples (Bieniak et al., 2024; Griffin et al., 2023) or focused on specific populations (Bailey et al., 2022; Derrer‐Merk et al., 2023; Macpherson et al., 2022). International ‘Big Qual’ studies found that long‐term wellbeing was substantially shaped by experiences of loss at the start of the pandemic (Albo et al., 2025; Lowe et al., 2024) further highlighting the need to explore such datasets to understand how wellbeing and behaviour can be supported over time.

Whilst data collection for our previous studies and the wider qualitative COVID literature was conducted within relatively narrow timeframes, the exploratory findings presented here offer temporal insight into how themes may have played out longitudinally by reporting the prevalence of generated topics at different timepoints (3, 6, 12 and 24 months post‐lockdown). For example, the topic of ‘Stress’ was most prevalent as England was emerging from the first lockdown (3 months), then decreased slightly over time. This aligns with studies that found that increases in mental health issues during the first UK lockdown subsequently declined as restrictions eased, although levels of psychological distress remained elevated compared to pre‐pandemic (Daly et al., 2020; Dhensa‐Kahlon et al., 2025; Fancourt et al., 2020). The increased prevalence of the topic of ‘Salutogenesis’ immediately after 2020 and 2021 hard lockdowns (3 and 12 months) likewise corresponds with literature indicating that these periods could facilitate purposeful engagement in meaningful, and sometimes new, activities to support physical and mental health (Ellen et al., 2021; Griffin et al., 2023; Hanson et al., 2023; Williams, 2021; Williams et al., 2021). Of particular note within our findings is the topic of concerns around ‘physical health’, an aspect of ‘disruption’ identified in our prior work (Hanson et al., 2023; Notley et al., 2022), with both topics in the theme initially having low prevalence, rising over time to have the highest prevalence at 24 months when nearly all restrictions had been lifted. While national UK statistics indicate relative stability in self‐reported general health over this period (Knight et al., 2024; Tan et al., 2024), other evidence relating to an ongoing backlog in health care service provision highlights the importance of continued surveillance of the longer‐term experience of secondary, negative health consequences (Derrer‐Merk et al., 2023; Zhai & Du, 2020).

### Strengths and limitations

A key strength of the study was its longitudinal design covering a period of significant social challenge and change, covering almost 2 years from when England was beginning to emerge from the first national lockdown to when nearly all restrictions had lifted. MATA enabled the rapid analysis of this large longitudinal qualitative dataset that would not have been feasible to analyse manually within the available resources. The reduced time required relative to manual analysis, which can be over four times as time consuming (Towler et al., 2023), demonstrates the method's ability to improve the efficiency of coding and theme generation. Furthermore, MATA's efficiency increases as datasets get larger, as larger datasets would not typically require any additional analysis time.

The analysis was conducted using a systematic and established method, MATA, which has been validated and used on other datasets (Bondaronek et al., 2023; Towler et al., 2023). This approach ensured rigorous implementation of the methodology and reliable interpretation of the results. Instead of relying on general‐purpose AI platforms, such as LLMs, which have been criticized for their lack of transparency and interpretability (Kornblith et al., 2022; Ziems et al., 2023) as ‘black‐box’ models, a bespoke topic modelling approach provided greater transparency and control. It enabled a collaborative process where experienced qualitative researchers and domain experts retained oversight and active involvement in the production of the findings, ensuring the themes were coherent and interpretable. Whilst providing researchers control over interpretation, what may be lost with this approach, however, are the ‘lightbulb moments’ in manual qualitative research where a single participant with a unique position or unusual context gives a perspective that illuminates understanding, advancing knowledge, from an N of 1 perspective.

While weighting metrics enabled a non‐biased method of allocating free‐text entries into a primary topic, some responses could plausibly fit into multiple topics, leading to occasional overlap and reducing specificity in interpretation. This highlights the need for caution in defining topic boundaries and ensuring nuanced analysis. The themes generated through MATA were consistent with findings from other qualitative studies in the C‐19 Health Behaviour and Wellbeing Daily Tracker Study (Hanson et al., 2023; Notley et al., 2022), supporting the validity of the findings and demonstrating the method's ability to produce meaningful insights.

Covariates can be included in the MATA analysis, allowing for quantitative exploration of the qualitative findings, as demonstrated in this paper by ranking and plotting mean prevalence scores by timepoint. This process could also be taken to understand how topics vary across participant characteristics (e.g., gender, deprivation), helping to detect patterns that might not be easily discernible in qualitative analysis alone. This mixed‐methods approach has the potential to increase generalizability and replicability of analysis and could assist in forming hypotheses about sociocultural influences. However, caution should be applied where it is used, as it could lead to erroneous conclusions. This is especially relevant when data relates to perspectives self‐reported by participants, rather than systematically measured phenomena. Our interpretation of the prevalence data was assisted by triangulating with previous study findings and the literature, and we advocate for triangulation where possible.

While MATA reduces the need for researcher reflexivity, it remains essential for researchers to critically engage with the interpretation of machine‐generated topics. This is particularly important given the rise of LLMs, where the potential for algorithmic bias is not only a concern but may be further exacerbated by these technologies (Hu et al., 2024; Zack et al., 2024). Developing reflexive practices tailored to machine‐assisted analysis is essential to address biases inherent in data and algorithms. To the authors' knowledge, no established methods currently exist to systematically guide this process, highlighting an urgent need for methodological development.

### Implications for research, practice, policy

MATA provides a practical solution for analysing large‐scale qualitative datasets quickly and rigorously while utilizing qualitative research expertise to extract meaning, interpret findings, work collaboratively and apply theoretical frameworks. Its application in this study demonstrates its potential to become a standard method for researchers working with Big Qual datasets, particularly in contexts where scalability and efficiency are critical (Brower et al., 2019; Chandrasekar et al., 2024). The broader applicability of MATA to diverse qualitative data types, such as in‐depth interview transcripts, is uncertain. Further investigation is required to determine its suitability for richer, less structured datasets.

The use of AI in qualitative analysis may challenge epistemological foundations of qualitative research by applying a reductionist approach to data analysis. This may be seen to be at odds with interpretivism, which emphasizes exploration of subjective meanings and constructed realities in analysis. Taking a pragmatist position, MATA was considered the best approach for this study, given the large longitudinal dataset, but need for flexibility in interpretation that human‐led analysis provides to ensure validity. Adopting a ‘human in control’ approach, such as MATA, may be more reflective of critical realism, acknowledging computer generated patterns in the textual data exist, but allowing for human exploration of conceptual meaning situated in the wider societal context. As technology advances, and human adoption of it increases, we may see new paradigms emerge however (Williams, 2024).

The quantification of qualitative data raises important considerations about balancing efficiency with the depth and quality of machine outputs. Researchers must critically evaluate how quantification impacts the interpretative process and ensure that the outputs are meaningful, nuanced and transparent. Whilst this process was undertaken through discussion for this study, as AI‐assisted qualitative analysis evolves and becomes more prevalent, it will become necessary to develop evaluation tools. A key step in this process involves the development of an evaluation framework tailored to machine‐assisted analysis methods, such as topic modelling. Such a framework would provide researchers with quality indicators for assessing AI‐generated outputs, ensuring that scalability does not come at the expense of interpretative rigour.

Reflexivity in Big Qual requires redefinition to address systemic biases inherent in data and algorithms. The use of AI approaches such as natural language processing, and in particular LLMs, in qualitative research brings new ethical challenges, particularly in ensuring the transparency, reliability and fairness of AI‐generated outputs. A quality evaluation framework that can be systematically applied to evaluate the quality of AI output of Big Qual data is needed. This would ensure that the findings remain ethical, nuanced and aligned with the principles of qualitative research, increasing the quality, transparency and trust in the utility of Big Qual methods.

AI models often prioritize dominant patterns in training data, which can lead to the amplification of majority viewpoints while marginalizing less prevalent perspectives (Peterson, 2024). This risk of underrepresenting minority voices highlights the need for intentional efforts to counteract bias. Incorporating user‐centred, participatory approaches should help ensure that AI tools are designed and applied in a way that is both equitable and ethically sound (Birhane et al., 2022).

## CONCLUSION

The application of MATA to qualitative free‐text data demonstrated its ability to efficiently identify meaningful patterns while maintaining researcher oversight. We identified key self‐reported factors influencing health behaviours, mood and wellbeing across different time points during the COVID‐19 pandemic. This study advances machine‐assisted topic analysis by demonstrating a hybrid approach that increases researcher control and interpretability. As AI continues to shape research methods, ensuring transparency and conceptual rigour remains critical. Future research should focus on developing systematic evaluation frameworks to ensure transparency, reliability and the mitigation of both machine‐ and human‐‘induced’ bias. Reflexivity is important in this process, particularly given the risks of algorithmic bias in AI‐driven methods. Additionally, participatory, user‐centred approaches are needed to ensure that AI‐driven qualitative analysis is applied ethically and equitably, with frameworks that integrate both evaluation and reflexivity to uphold the rigour of qualitative research. By incorporating covariate analysis, MATA exemplifies how computational approaches can complement qualitative methodologies, offering a more integrated mixed‐methods framework that preserves depth while enabling insights into different patterns and groups, especially those who are marginalized.

## AUTHOR CONTRIBUTIONS

**Emma Ward:** Conceptualization; methodology; validation; formal analysis; supervision; writing – review and editing; writing – original draft; investigation; visualization; data curation; project administration. **Felix Naughton:** Conceptualization; investigation; funding acquisition; writing – original draft; writing – review and editing; methodology; formal analysis; project administration; validation; data curation. **Pippa Belderson:** Writing – review and editing; formal analysis; writing – original draft; methodology. **Trisevgeni Papakonstantinou:** Investigation; software; formal analysis; data curation; methodology; writing – review and editing; writing – original draft. **Ben Ainsworth:** Conceptualization; writing – review and editing; data curation; formal analysis; methodology; investigation; validation. **Sarah Hanson:** Writing – review and editing; formal analysis; investigation. **Caitlin Notley:** Funding acquisition; writing – review and editing. **Paulina Bondaronek:** Conceptualization; investigation; writing – original draft; writing – review and editing; methodology; validation; formal analysis; software; data curation; supervision.

## Supporting information


Appendix S1.

## Data Availability

The data that support the findings of this study are available from the corresponding author upon reasonable request. The data are not publicly available due to privacy or ethical restrictions.
