# Supplementary figures and images for: Using machine‐assisted topic analysis to expedite thematic analysis of free‐text data: Exemplar investigation of factors influencing health behaviours and wellbeing during the COVID‐19 pandemic
Source: Br J Health Psychol. 2025 Sep 11;30(3):e70017. doi: 10.1111/bjhp.70017 (PMC12426459; doi:10.1111/bjhp.70017)

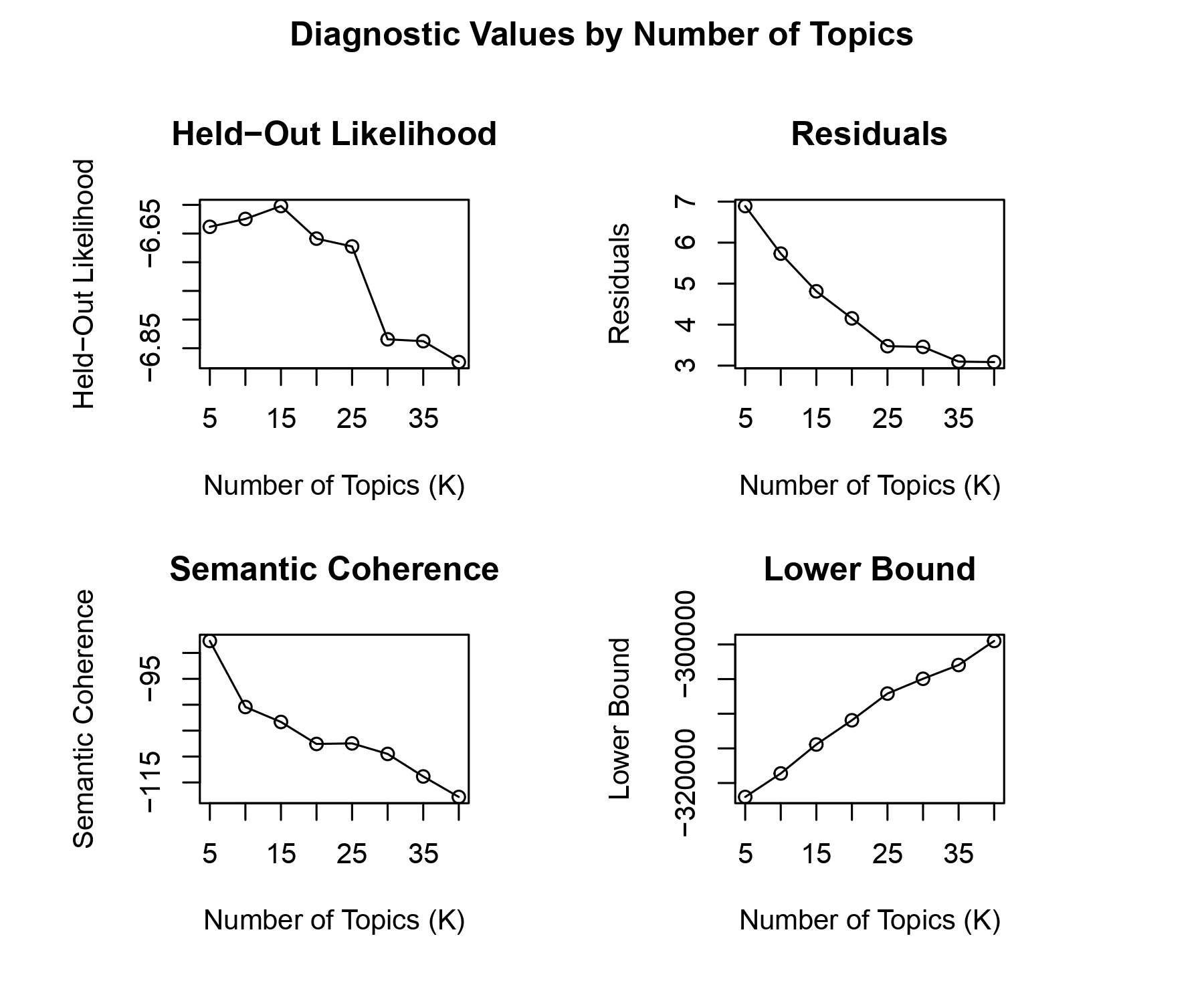

Supplement: Supplementary file 1 — Appendix S1. [file BJHP-30-0-s001.jpg]
